# 1-Deoxynojirimycin Ameliorates Diabetic Liver Injury by Regulating AMPK/SIRT1 and Oxidative Stress in db/db Mice

**DOI:** 10.2174/0118715303327499250104221937

**Published:** 2025-02-19

**Authors:** Yao Li, Yu Cao, Yun-Yuan Tian, Wen-Wen Chen, Juan Wang, Meng-Meng Zhang, Si-Wang Wang, Yan-Hua Xie

**Affiliations:** 1 The College of Pharmacy, Shaanxi University of Chinese Medicine, Xianyang 712046, China;; 2 Department of Chinese Materia Medica and Natural Medicines, Air Force Medical University, Xi’an 710032, China;; 3 The College of Life Sciences, Northwest University, Xi’an 710069, China

**Keywords:** 1-DNJ, diabetic liver injury, steatosis, fibrosis, AMPK/SIRT1 signaling pathway, oxidative stress

## Abstract

**Background:**

Patients with diabetic liver injury are in the dilemma of lowering glucose and protecting liver function. This study aimed to uncover the protective effect and mechanism of 1-deoxynojirimycin (1-DNJ), an alpha-glucosidase inhibitor, against diabetic liver injury.

**Methods:**

The db/db mice were gavaged with 25 mg/kg, 50 mg/kg, and 100 mg/kg of 1-DNJ for 8 weeks. At the end of the administration, the serum and liver were isolated for further detection. The biochemical indices including TC, TG, LDL-C, AST, ALT, and TBiL were detected in serum. The livers were further analyzed with H&E, oil red, and Masson staining, and the amount of ROS in the liver was detected with probe dihydroethidium. Western blot was used to analyze the levels of proteins involved in fibrosis, oxidative stress, and AMPK/SIRT1 signaling pathway in the liver.

**Results:**

1-DNJ administration reduced body weight, liver coefficient, and TC, TG, LDL-C, AST, ALT, and TBiL in db/db mice. H&E and oil red staining showed that 1-DNJ ameliorated hepatocellular ballooning degeneration and lipid deposition in the liver. Moreover, 1-DNJ reduced the hepatic collagen fiber deposition and the protein expression of α-SMA and Collagen I. Further assays revealed that 1-DNJ treatment reduced the ROS level, up-regulated the proteins expression of SOD2, HO-1, NQO-1, p-AMPK/AMPK, p-ACC/ACC, and SIRT1 proteins, and down-regulated the expression of SREBP-1 and SCD-1 proteins in the liver.

**Conclusion:**

1-DNJ improves liver function, lipid deposition, and fibrosis of diabetic liver injury in db/db mice by regulating the AMPK/SIRT1 pathway to improve glucose-lipid metabolism and oxidative stress.

## 1 INTRODUCTION

Diabetes has become one of the most common metabolic diseases that causes muti-organ complications such as cardiopathy, nephropathy, hepatopathy, retinopathy, and neuropathy, counting 529 million diabetic people worldwide in 2021 [1, 2]. Diabetic liver injury is mainly characterized by elevated alanine aminotransferase (ALT), aspartate aminotransferase (AST), steatosis, and fibrosis of the liver, also known as diabetes combined with nonalcoholic fatty liver disease (NAFLD) or nonalcoholic steatohepatitis (NASH) [3, 4]. A series of studies found that the prevalence of NAFLD among type 2 diabetes mellitus (T2DM) patients was 55.5%, and the prevalence reached 68.0% in Europe [5]. T2DM is the most important risk factor and significant predictor of adverse clinical outcomes for NAFLD and NASH, and the risk of hepatic decompensation and hepatocellular carcinoma in people with T2DM at 5 years was 13.85% and 3.68%, respectively [6, 7].

AMP-activated protein kinase (AMPK), as the main energy receptor in the body, is a regulator of hepatic energy metabolism [8]. AMPK regulates a variety of physiological processes related to metabolic regulation through the activation of sirtuin 1 (SIRT1), such as liver glucose-lipid metabolism, muscle tissue differentiation, and adipose tissue formation [9]. Acetyl-CoA carboxylase (ACC) as a key regulator of lipid synthesis, AMPK directly phosphorylates ACC1 and ACC2 to produce malonyl-coenzyme A in the outer mitochondrial membrane, thus inhibiting fatty acid synthesis and promoting fatty acid oxidation. Up-regulation of AMPK further inhibits the expression of sterol regulatory element binding protein 1 (SREBP1) and stearoyl-CoA desaturase 1 (SCD-1), which in turn acts on the transcription and expression of ACC, thus jointly participating in hepatic lipid synthesis. The dysfunction of AMPK resulting in an imbalance of energy metabolism can directly trigger T2DM, which leads to abnormal accumulation of hepatic fatty acids and forms the first strike to the organism [10]. In addition, lipid peroxidation due to oxidative stress induced by reactive oxygen species (ROS) can cause a second hit to the organism, thus aggravating the development of diabetic liver injury [11]. SIRT1 can be activated not only by AMPK, but also deacetylated to activate AMPK and protect the liver from oxidative stress at the same time [12]. AMPK/SIRT1 pathway activation can simultaneously attenuate lipo-toxicity and oxidative stress-induced liver damage and is one of the important targets for the treatment of diabetic liver injury.

**Fig. (1):**
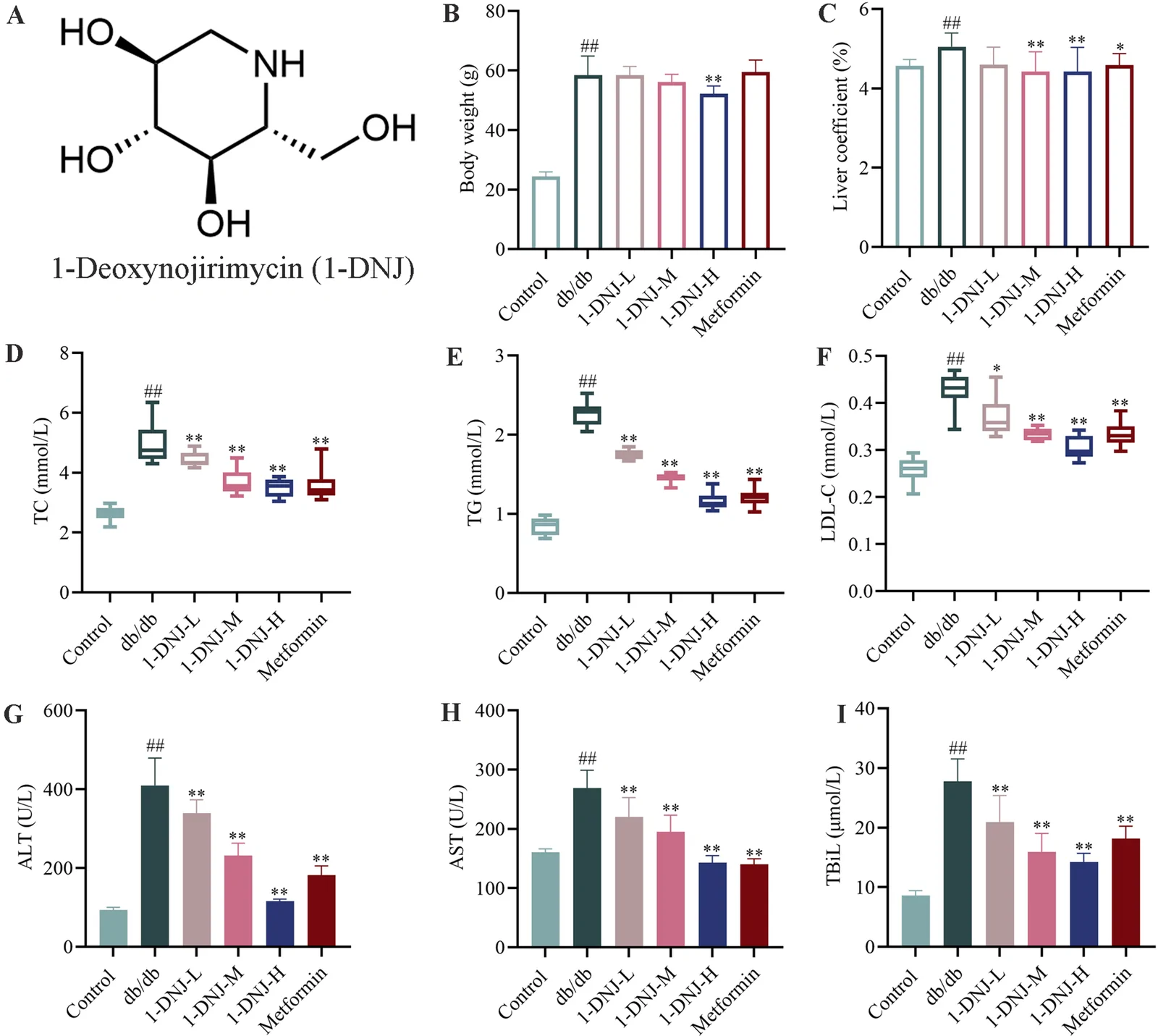
1-DNJ improves serum lipids and liver function in db/db mice. (**A**) The chemical structure of 1-DNJ. (**B**) Body weight of mice at 16 weeks. (**C**) Liver coefficient. (**D**) The TC content in serum. (**E**) The TG content in serum. (**F**) The LDL-C content in serum. (**G**) The ALT content in serum. (**H**) The AST content in serum. (**I**) The TBiL content in serum. N = 8. ^##^*P* < 0.01 *vs.* Control group; *P < 0.05, **P < 0.01 *vs.* db/db group. N=10.

1-Deoxynojirimycin (1-DNJ) as one of the main active components of mulberry leaves in lowering blood glucose (Fig. **1A**), is a natural alkaloid that can inhibit the α-glucosidase activity to improve postprandial blood glucose and insulin resistance and inducing the risk of T2DM in hyperlipidemic mice [13, 14]. Meanwhile, intravenous injection of 1-DNJ regulates the blood glucose by modulating the expression of glucose metabolism rate-limiting enzymes in the liver to reduce glucose production and also promotes lipolysis and attenuates the number of hepatic lipid droplets in diabetic mice [15]. However, the mulberry leaves used for the treatment of diabetes were always administrated orally, so whether 1-DNJ taken orally also has a protective effect on diabetic liver injury, and the mechanism of 1-DNJ in alleviating hepatic lipid deposition and ameliorating diabetic liver injury in diabetic mice remains uncovered. It has been reported that mulberry leaf flavonoids improve T2DM by regulating the AMPK/SIRT1/PGC-1α pathway, and the previous study of our group also found that mulberry leaf and its main component 1-DNJ can improve myocardial injury in diabetic mice by regulating SIRT1 [16]. However, whether 1-DNJ ameliorates diabetic liver injury is related to the regulation of the AMPK/SIRT1 pathway remains to be elucidated.

Spontaneous T2DM db/db mice are ideal animal models for studying diabetic liver injury due to the early elevation of blood glucose, defective insulin function, and the development of significant liver damage including hepatocellular steatosis and inflammatory cell infiltration at 16 weeks of age [17]. Therefore, in this study, db/db mice were employed and given different doses of 1-DNJ until 16 weeks of age, the hepatic function, lipid deposition, and hepatic fibrosis were detected, and it was found that 1-DNJ alleviated the liver impairment in db/db mice. Further study revealed that 1-DNJ ameliorated diabetic liver injury by modulating the AMPK/SIRT1 pathway and oxidative stress.

## 2 MATERIALS AND METHODS

### 2.1 Animals

Fifty specific pathogen-free BKS-db and ten BKS male mice, aged 4-6 weeks and weighing 18-24 g, were purchased from Gempharmatech Company (License No: SCXK (Chuan) 2020-034). All mice were fed with a standard diet and kept in the environment with alternating light day and night of 12 h, temperature of 23-25°C, and relative humidity of 60%-70%. The study was implemented after mice were acclimated for 2 weeks.

### 2.2 Animal Treatment

The BKS-db mice were randomly divided into five groups (n=10): the model group (db/db group), the 1-DNJ low dose group (1-DNJ-L), the 1-DNJ medium dose group (1-DNJ-M), 1-DNJ high dose group (1-DNJ-H), and positive control group (Metformin). The ten BKS mice were assigned to a control group. The mice in the 1-DNJ-L, 1-DNJ-M, and 1-DNJ-H group were administered with 1-DNJ (HI106902, ≥ 98%, Herbest, Baoji, China) at dose of 25 mg/kg, 50 mg/kg, and 100 mg/kg, and mice in the metformin group were gavaged with metformin (B25331, ≥ 98%, Shanghai yuan ye Bio-Technology, Shanghai, China) at dose of 200 mg/kg for consecutive 8 weeks, while mice in the control group and db/db group received the equal volume deionized water (Determination of treatment time after assessment of mental status and coat color in mice). At the end of the experiment, the mice were anesthetized with sodium pentobarbital, the blood was collected to analyze biochemical indicators in serum, and the liver was separated and weight to calculate the liver coefficient as the following formula. Then the liver was immersed in 4% paraformaldehyde or stored at -80°C for further testing.

Liver coefficient (%) =liver weight (g) / body weight of mice at 16 weeks (g) ×100%

### 2.3 Detection of TC, TG, and LDL-C Content in Serum

The blood samples were centrifuged at 3500 rpm 4°C for 15 min to obtain serum, and the total cholesterol (TC), triglyceride (TG), and Low-density lipoprotein cholesterol (LDL-C) were detected by employing the TC assay kit (A111-1-1, Nanjing Jiancheng Bioengineering Institute), TG assay kit (A110-1-1, Nanjing Jiancheng Bioengineering Institute), and LDL-C assay kit (A113-1-1, Nanjing Jiancheng Bioengineering Institute). 250 μL working solution of TC or TG was added to the 96-well plate, and 2.5 μL serum, calibrator, or distilled water, respectively. The optical density (OD) was detected at 500 nm after incubation at 37°C for 10 min, and the TC and TG contents were calculated according to the formula: TC or TG content = (OD _samples_ - OD _blank_) / (OD _standard_ - OD _blank_) × C _standard_. 180 μL LDL-C reagent1 was added to the 96-well plate, and following adding 2.5 μL serum, calibrator, or distilled water, the OD was detected and recorded as A1 at 600 nm after incubation at 37°C for 5 min, then 60 μL reagent2 was added in each well and detected OD (recorded as A2) at 600 nm after incubation at 37°C for 5 min. The content of LDL-C was calculated as follows: LDL-C content = (ΔA _sample_ - ΔA _blank_) / (ΔA _standard_ - ΔA _blank_) × C _calibration_; ΔA = A2 -A1.

### 2.4 Detection of ALT, AST, and TBil Content in Serum

The content of alanine aminotransferase (ALT), aspartate aminotransferase (AST), and total bilirubin (TBiL) in serum were detected according to the applicable recommendations of ALT assay kit (C009-2-1), AST assay kit (C010-2-1), and TBiL assay kit (C019-1-1) purchased from Nanjing Jiancheng Bioengineering Institute (Nanjing, China).

### 2.5 H&E Staining

The liver fixed in 4% paraformaldehyde was embedded in paraffin blocks cut into 3-5 μm slices, and stained by Hematoxylin and Eosin Staining Kit (C0105S, Beyotime, Shanghai, China). The slice was deparaffinized twice in xylene for 8 min each time and then put into 100%-70% gradient ethanol for 2 min respectively. After the wax slices were processed, the slices were stained with Hematoxylin Stain for 10 min, and rinsed in tap and distilled water for 10 min, and then stained with Eosin Stain for 1 min. The images were observed by a Nikon Eclipse E100 microscope after dehydrated, transparent, and sealed.

### 2.6 Oil Red O Staining

The fresh liver was sliced up to 4-8 μm by frozen section and rewarm for 10 min in the sectioning rack. A staining washing solution was added to the slices to cover the samples for 20 seconds. After aspirating the washing solution, the slices were immersed into oil red O staining working solution (oil red O solution: oil red O dilution = 3: 2) for 20 min, and after the staining was completed the slices were added washing solution and rest for 30 s. Then the slice was placed into the shaking bed of distilled water to be washed for 20 s and stained with hematoxylin to re-stain the nuclei. The slice was observed by a Nikon Eclipse E100 microscope after sealed.

### 2.7 Masson Trichrome Staining

The paraffin slices of the liver tissue were stained with 50 μL hematoxylin staining solution for 5 min, and after removing the staining solution, the slice was washed with distilled water for 5 min, and acidic differentiation solution was added to differentiate for 30 s, and rinsed with tap water to return blue. Then 50 μL Ponceau's-acid fuchsin staining solution was added to stain the sections for 10 min, and phosphomolybdic acid differentiation solution was added to differentiate the sections for 2 min, and then the sections were stained with 50 μL Brilliant Green staining solution for 1 min, and dehydrated and sealed the sections for image acquisition with the Nikon Eclipse E100 microscope.

### 2.8 Detection of ROS Level

Dissolve 1 μL of ROS probe dihydroethidium in 500 μL of diluent as ROS working solution. The 4-8 μm frozen section of the liver was washed with phosphate buffer solution for 5 min to remove the embedding agent, and incubated for 30 min at 37°C in the dark with ROS working solution. Then the nucleus was stained with a DAPI staining solution, and the fluorescence intensities were measured at λex/λem=520/605 nm with a Nikon Eclipse Ti confocal microscope.

### 2.9 Western Blot

Proteins from the liver tissue of the mice were separated by SDS electrophoresis and transferred to PVDF membranes. The following primary antibodies were used to incubated overnight with membranes at 4°C: anti-AMPK (WL02254), anti-p-AMPK (WL02254) (Wanleibio, Shenyang, China); anti-α-SMA (AF1032), anti-CollagenI (AF7001),, anti-ACC (AF6421), anti-p-ACC (AF3421), anti-SIRT1 (DF6033), anti-SREBP1 (AF6283), anti-SCD-1 (DF13253), anti-Keap1 (AF5266), anti-HO-1 (AF5393), anti-SOD2 (AF3751), anti-NQO-1 (DF6437) (Affinity Biosciences, OH, USA); anti-β-actin (GB15001) (Servicebio, Wuhan, China) was used as the internal control. Then the membranes were treated with a secondary antibody for 1.5 h after washing away the primary antibody with TBST. The intensity of each protein band was scanned and analyzed by ChemiDoc™XRS + Imaging System.

### 2.10 Statistics

All data were analyzed using IBM SPSS 23.0 (Armonk, NY, United States). One-way analysis of variance (ANOVA) was performed after the measurement of homogeneity of variance, and post hoc comparisons with the least significant difference (LSD) test were used to compare the data between different groups. The data are presented as the mean ± standard deviation, with *P* < 0.05 considered statistically significant. (Supplementary material)

## 3 RESULTS

### 3.1 1-DNJ Improves Serum Lipids and Liver Function in db/db Mice

Diabetic liver injury is mainly manifested as the abnormalities of glucose-lipid metabolism and liver function. As shown in Fig. (**1**), compared with the control group, the body weight, TC, TG, and LDL-C in serum was elevated, and further detection of liver function showed that the liver coefficient, ALT, AST, and TBiL were also increased in serum in db/db mice at 16 weeks, indicating that the abnormal lipid metabolism and impaired hepatic function of db/db mice (*P* < 0.01). Interestingly, administrating different doses of 1-DNJ and metformin decreased the body weight and serum lipids including TC, TG, and LDL-C in the serum of db/db mice. Moreover, 1-DNJ also improved liver function, such as decreasing the ALT, AST, and TBiL in the serum of db/db mice, and the effect of 1-DNJ-H was the most significant (*P* < 0.01).

### 3.2 1-DNJ Alleviates the Lipid Deposition of the Liver in db/db Mice

**Fig. (2):**
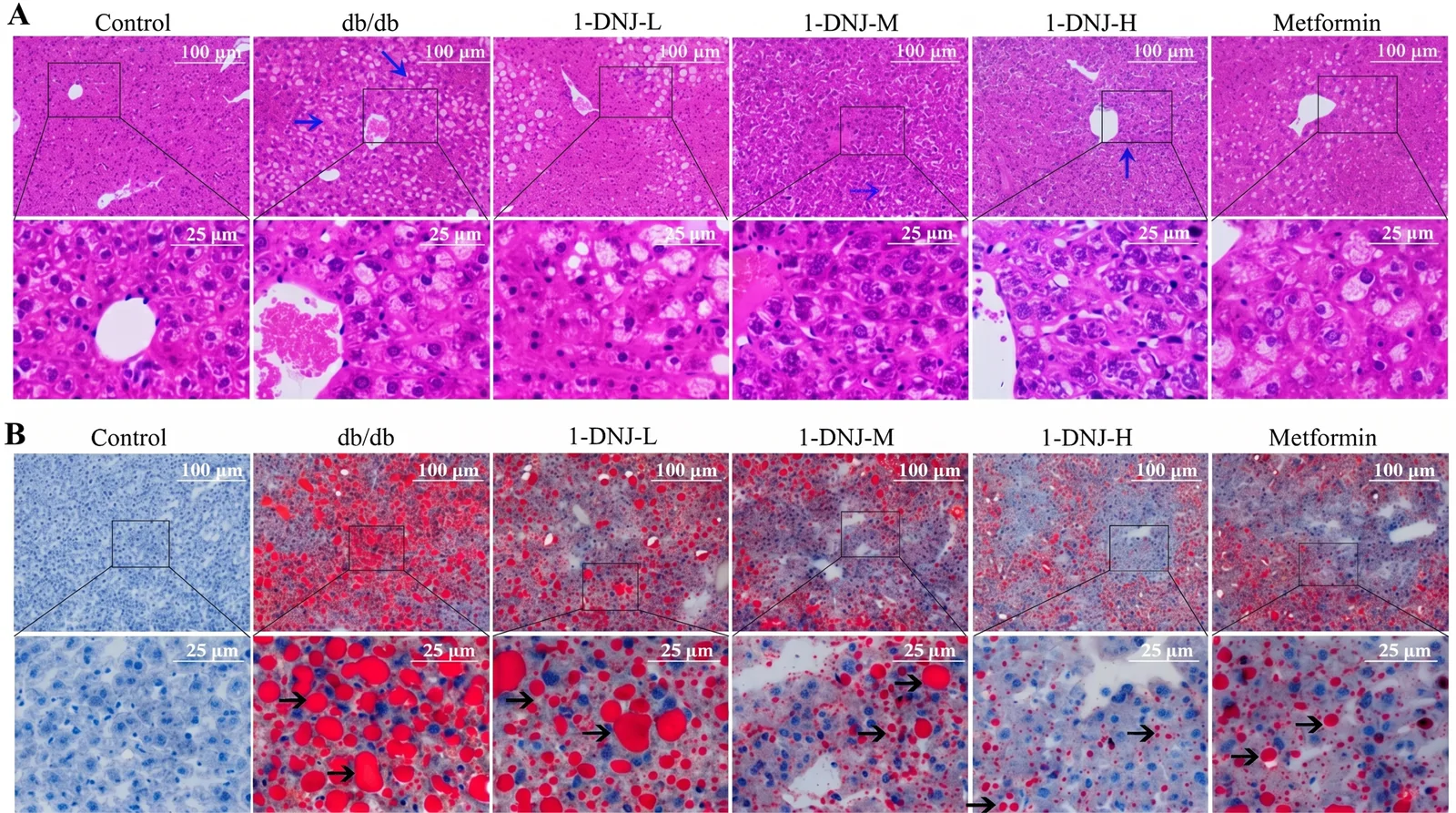
1-DNJ alleviates the lipid deposition of the liver in db/db mice. (**A**) Representative H&E staining of hepatic tissue in each group. (**B**) Representative Oil Red O staining of hepatic tissue in each group. N = 3. **→** represents ballooning degeneration area; **→** represents the lipid droplets.

The lipid deposition is the liver is a main cause of diabetic liver injury. H&E staining revealed that the hepatocyte appeared swelling, changed in morphology, and ballooning degeneration in db/db mice (Fig. **2A**). Oil-red staining further confirmed that a large number of lipid droplets were deposited in the livers of db/db mice (Fig. **2B**). In the 1-DNJ-L group, the liver cell swelling was relieved, but a large number of lipids droplet depositions persisted. While in the 1-DNJ-M and 1-DNJ-H group, the ballooning degeneration of liver cells and lipid droplet deposits were reduced significantly, and some hepatocytes were restored to normal, which suggests that 1-DNJ improves the hepatic lipid deposition in db/db mice.

### 3.3 1-DNJ Prevents the sSteatosis Development of the Liver in db/db Mice

**Fig. (3):**
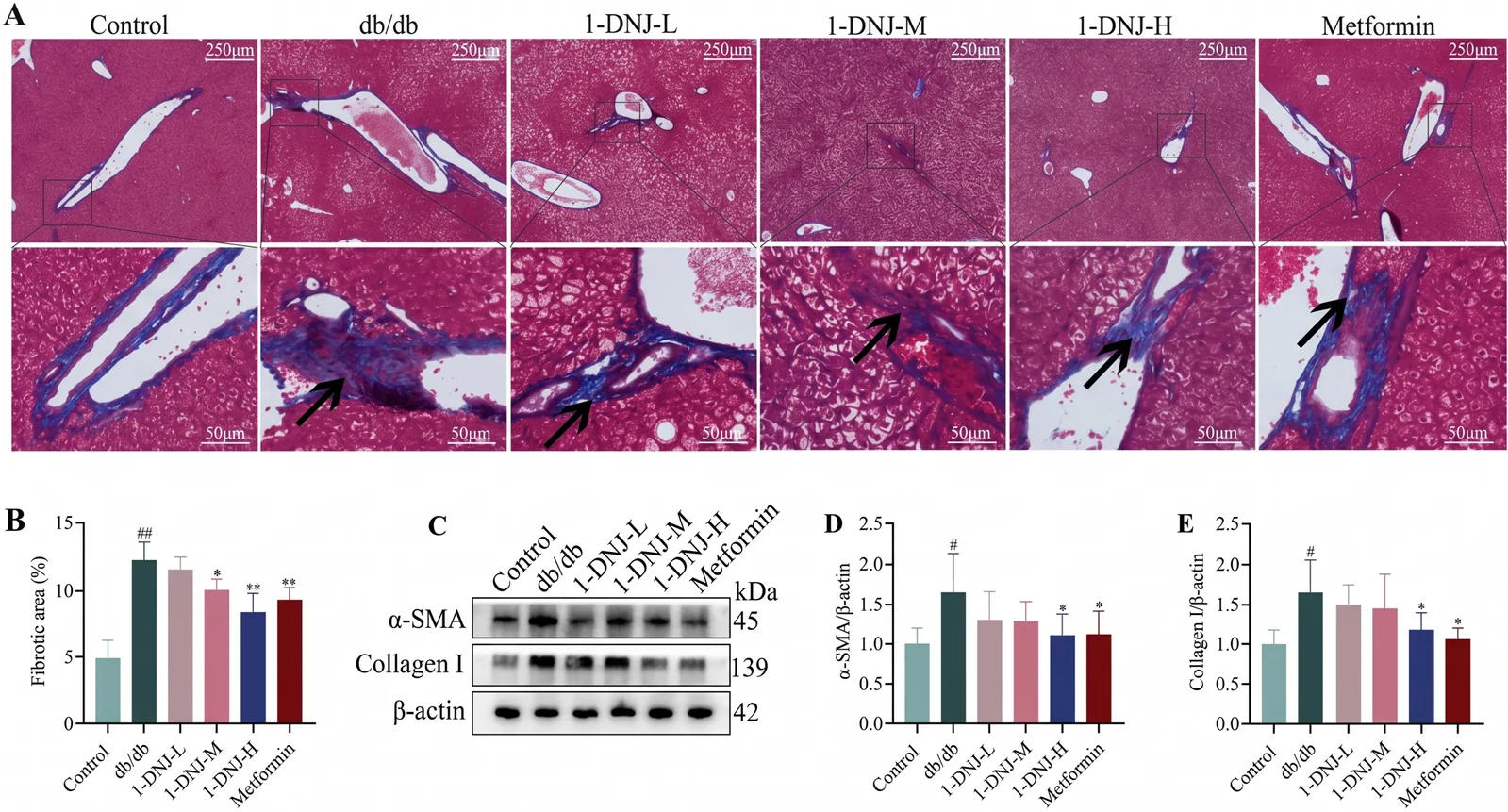
1-DNJ prevents the steatosis development of the liver in db/db mice. (**A**) Representative Masson staining of hepatic tissue in each group. (**B**) The fibrotic area of liver tissue. (**C**) The protein level of α-SMA and Collagen I in the liver was detected by western blotting, with gray intensity analysis shown in (**D**) and (**E**). N = 3. ^##^*P* < 0.01 *vs.* Control group; *P < 0.05, **P < 0.01 *vs.* db/db group. **→** represents the fibrous area.

Liver steatosis progression to NASH can further develop into liver fibrosis and cirrhosis. We further detected the fibrosis of the liver by Masson staining and western blotting, the results found that compared with the control group, db/db mice exhibited obvious collagen fiber deposition in multiple portal vein regions of the liver at 16 weeks (Fig. **3A**), with increased percentage of fibrotic regions (Fig. **3B**), and elevated protein expression of α-SMA and collagen I (Figs. **3C**-**E**, Fig. **S1**), suggesting that the diabetic liver injury progressed toward fibrosis in db/db mice. Compared with the db/db group, the collagen fiber in the portal vein and fibrotic area were not attenuated in the livers of mice in the 1-DNJ-L group, but with a tendency to reduce α-SMA and collagen I protein expression although not statistically different (*P* > 0.05). While the deposition of collagen fiber and fibrotic areas was attenuated in the livers of mice in the 1-DNJ-M and 1-DNJ-H group, and 1-DNJ also reduced the protein expression ofα-SMA and collagen I significantly (*P* < 0.05).

### 3.4 1-DNJ Mitigates the Oxidative Stress of the Liver in db/db Mice

**Fig. (4):**
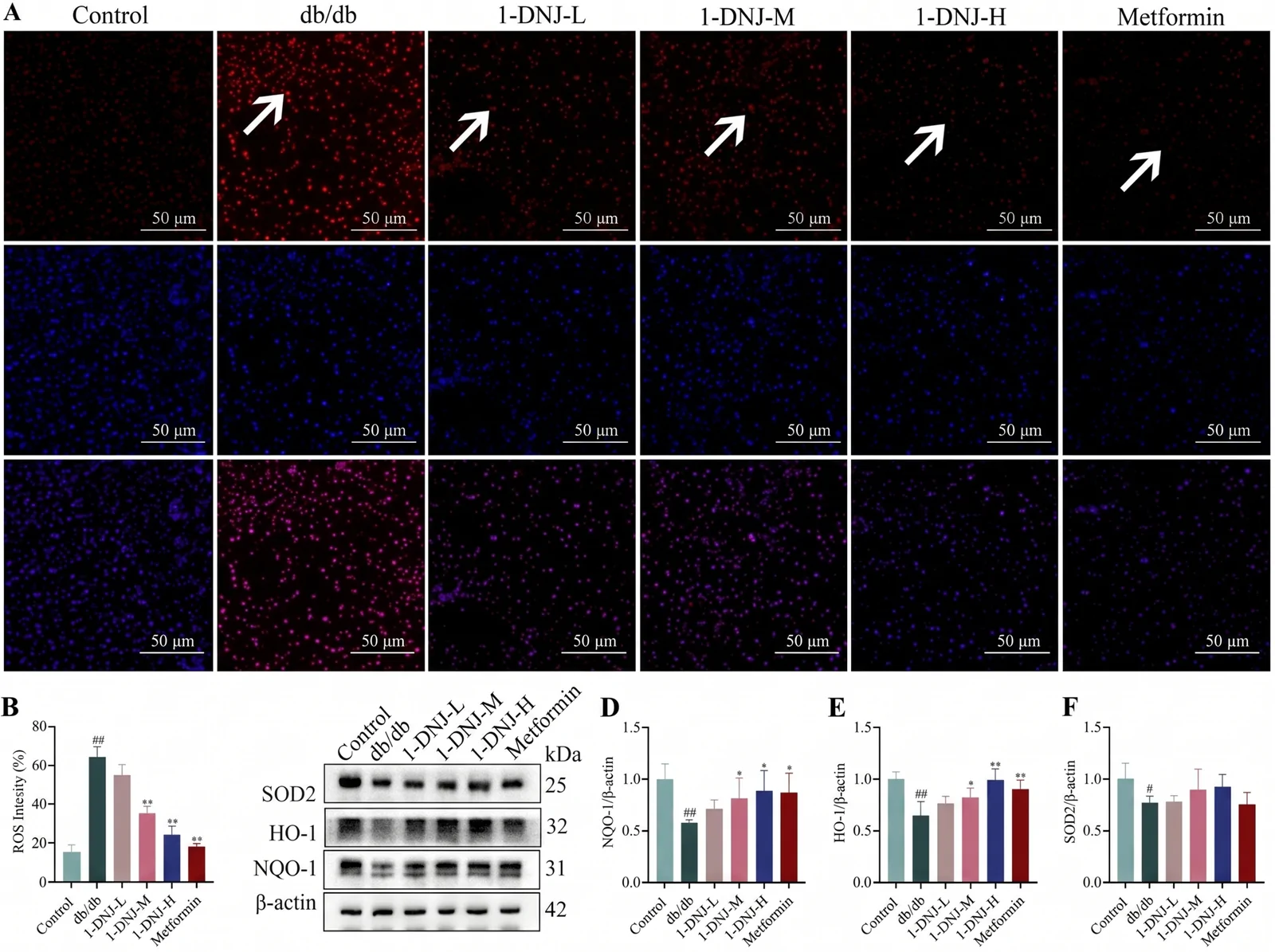
1-DNJ mitigates the oxidative stress of the liver in db/db mice. (**A**) Representative ROS fluorescence detected by dihydroethidium probe, with ROS intensity analysis shown in panel (**B**). (**C**) The protein expression of SOD2, HO-1, and NQO-1 in the liver was detected by western blotting, with gray intensity analysis shown in (**D-F**). N = 3. ^##^*P* < 0.01 *vs.* Control group; *P < 0.05, **P < 0.01 *vs.* db/db group. The red-colored fluorescent area indicated by the white arrow represents the intensity of ROS, and the stronger the red fluorescence, the more ROS.

Reactive oxygen species (ROS) generated by oxidative stress are an important contributor to liver fibrosis. Hence, the ROS in the liver was detected, which revealed that the ROS fluorescence intensity was increased in the liver of mice in db/db group, and 1-DNJ administrating reduced the ROS level in the liver, with 1-DNJ-M and 1-DNJ-H showed a significant difference compared with the db/db group (Figs. **4A**, **B**). Furthermore, the expression of anti-oxidative stress proteins including SOD2, HO-1, and NQO-1 was decreased in the liver of db/db mice, 1-DNJ-M and 1-DNJ-H increased the protein expression of HO-1 and NQO-1 but had no effect on SOD2 protein expression (Figs. **4C**-**F**, Fig. **S2**).

### 3.5 1-DNJ Regulating the AMPK/SIRT1 Pathway in db/db Mice

**Fig. (5):**
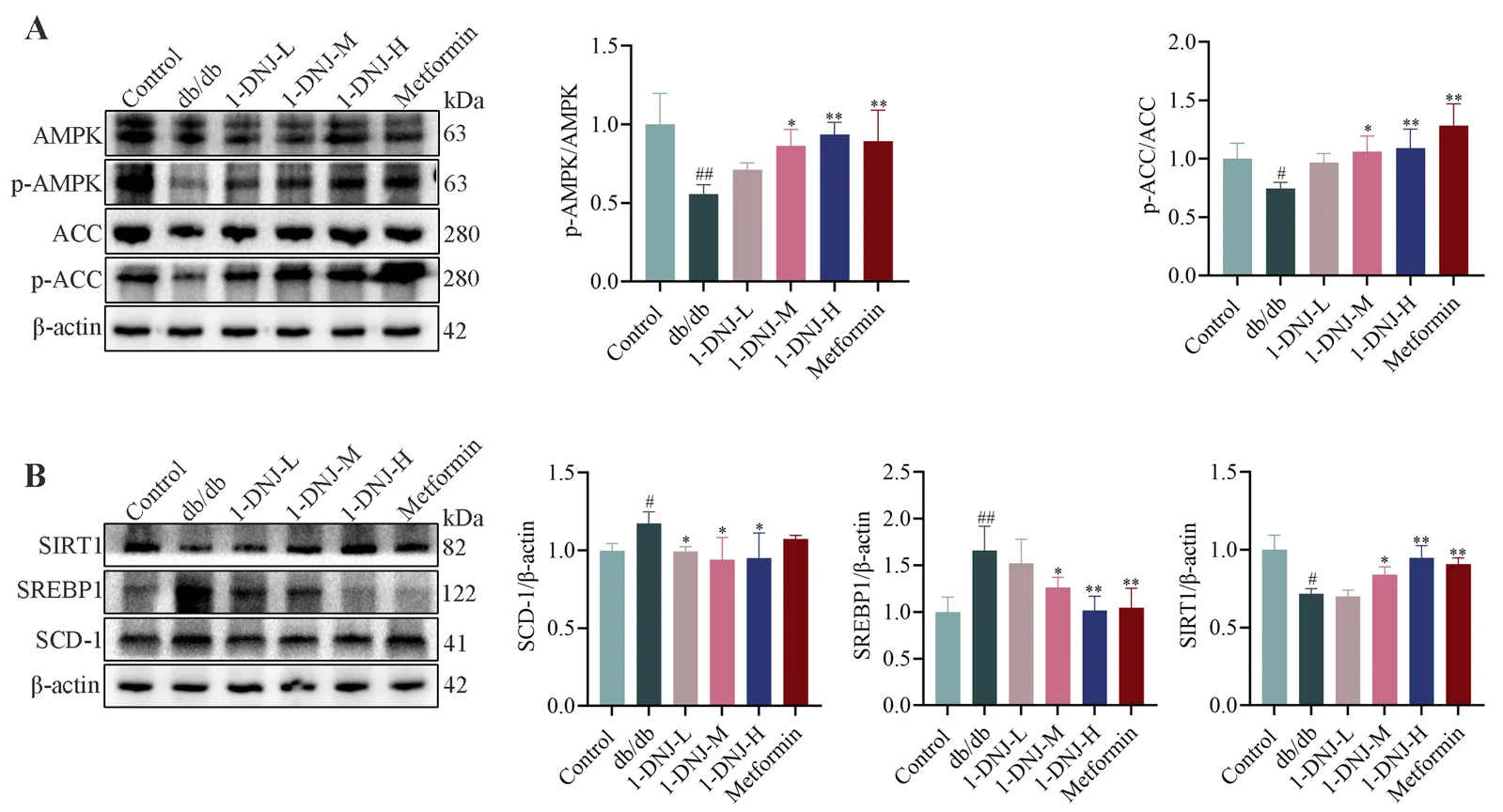
1-DNJ regulating the AMPK/SIRT1 pathway in db/db mice. (**A**) The protein expression of AMPK, p-AMPK, ACC, and p-ACC in the liver was detected by western blotting. (**B**) The protein expression of SIRT1, SREBP1, and SCD-1 in the liver was detected by western blotting. N = 3. ^##^*P* < 0.01 *vs.* Control group; *P < 0.05, **P < 0.01 *vs.* db/db group.

AMPK/SIRT1 is a classical upstream signaling pathway of oxidative stress and an important regulator of mitochondrial energy metabolism. Detection of AMPK and its downstream direct substrate ACC in mice's liver revealed that the protein expression of p-AMPK/AMPK and p-ACC/ACC was reduced in the liver of db/db mice, which indicated that the AMPK pathway was inhibited. Administrating of 1-DNJ-M and 1-DNJ-H upregulated the protein expression of p-AMPK/AMPK and p-ACC/ACC compared with mice in the db/db group (Fig. **5A**, Fig. **S3A**). Further investigation of the downstream proteins revealed that the protein expression of SIRT1 was downregulated, and SREBP1 and SCD-1 were upregulated in the liver of mice in db/db group (Fig. **5B**, Fig. **S3A**). While 1-DNJ treatment increased the SIRT1 protein expression, and decreased the SREBP1 and SCD-1 proteins expression at the same time.

## 4 DISCUSSION

The liver is the major organ regulating glucose metabolism, and its injury could in turn cause or exacerbate diabetes. Therefore, the treatment for patients with diabetic liver injury will face more challenges than that of patients with diabetes alone. Although there is a variety of hypoglycemic agents that effectively control glycemic levels in T2DM patients, metformin and acarbose that are not metabolized through the liver are the limited choice for the patients with diabetic liver injury. Sulfonylureas and repaglinide which are mainly excreted by the liver will further aggravate the liver injury [18, 19]. However, even metformin, acarbose, and pioglitazone, which are considered safer, are still contraindicated in diabetic patients who develop a severe liver injury or ghrelin exceeds 2.5-3 times the normal value [20]. Therefore, diabetic liver injury patients are faced with the dilemma of both lowering glucose and protecting liver function, and there is an urge for the development of effective therapeutic drugs that can both reduce blood glucose and protect the liver in patients with diabetic liver injury.

Diabetic was known as “XiaoKe Disease” in traditional Chinese medicine (TCM), and TCM has accumulated a wealth of experience in treating diabetes and its complications including diabetic liver injury after thousands of years of practice [21]. The mulberry leaf was recorded as “decoction instead of tea, can cure diabetes”, which was called “fairy leaf” in the “Compendium of Materia Medica”. The dictionary of Traditional Chinese medicine also states that “mulberry leaves have an antidiabetic effect” [22]. More importantly, mulberry leaves can protect liver function and reduce acute liver injury by improving the activity of liver metabolic enzymes and enhancing liver metabolism [23, 24]. 1-DNJ, a major constituent of mulberry leaves, has the same hypoglycemic and hepatoprotective effects as mulberry leaves [25]. In the study by Liu. *et al.*, the hepatic glucose metabolism was increased to further reduce the lipid deposition in the livers of diabetic mice by intravenous injection of 1-DNJ [15]. Interestingly, we found that oral administration of 1-DNJ similarly reduced the lipid deposition and elevated the ALT and AST levels to improve the liver function in the livers of db/db mice.

Liver fibrosis is the only pathological indicator of adverse liver outcomes, and the incidence of cirrhosis in NAFLD was only 0.6%-3% within 10-20 years, while the incidence of cirrhosis will rise to 15%-25% in patients with NASH within 10-15 years [26]. In patients with diabetic liver injury who have a combination of T2DM and NAFLD or NASH, excessive accumulation of lipids in the liver contributes to the development of a hypoxic microenvironment and chronic low-grade inflammation due to the coexistence of metabolic dysfunction and impaired hepatocyte function [27]. Chronic low-grade inflammation triggers the secretion of inflammatory factors, such as IL-1β, TNF-α, macrophage-inducible C-type lectin, and adiponectin, and the secretion of inflammatory modulators affects the production and degradation of extracellular matrix components [28]. Furthermore, the expression of key fibrosis proteins such as collagen I, collagen IV, matrix metalloproteinases, and α-SMA was upregulated to induce the deposition of hepatic extracellular matrix and the progression of fibrosis [29]. Our results showed that db/db mice exhibited significant lipid accumulation, increasing collagen fiber deposition around the portal vein, and elevating protein expression of collagen I and α-SMA in the liver at 16 weeks, while 1-DNJ ameliorated the fibrosis in the livers of db/db mice.

Oxidative stress is an important culprit in the progression of hepatic steatosis to fibrosis in patients with diabetic liver injury [30]. Normally, mitochondria in the liver convert 2% of consumed O_2_ to ROS, and with the deposition of lipid droplets in hepatocytes of patients with diabetic liver injury, ATP production is reduced in damaged hepatocytes, and abnormal energy metabolism altered the processes of mitochondrial fission and fusion. Mitochondrial dysfunction further leads to increased ROS production and the generation of a pro-inflammatory and immunosuppressive microenvironment and activates the apoptotic and necrotic pathway that significantly contributes to the progression of hepatic steatosis toward fibrosis [31]. It is reported that the damage of mitochondrial DNA and mitochondrial respiratory chain proteins accompanied by excessive ROS production was detected in NAFLD patients, and NASH patients in progression also exhibited evident elevated ROS levels and reduced catalase activity [32]. Consistently, elevated ROS levels and decreased antioxidant proteins such as NQO-1, HO-1, and SOD2 were observed in the liver of db/db mice at 16 weeks, and 1-DNJ administration alleviated the oxidative stress by reducing the hepatic ROS production and up-regulating the expression of antioxidant proteins in db/db mice in our study.

AMPK/SIRT1 signaling pathway plays a critical role in the pathogenesis of diabetic liver injury and is an important target for the treatment of T2DM and its complications [33]. Activation of the AMPK/SIRT1 pathway not only modulates hepatic glucose-lipid metabolism by inhibiting fat accumulation and free fatty acid levels but also reduces cellular oxidative stress by inhibiting intracellular NADPH oxidase-derived ROS damage [34]. The oxidative stress will be exacerbated by inhibition of the AMPK/SIRT1 pathway, and oxidative stress in turn further down-regulates the protein expression of AMPK and SIRT1 in a vicious cycle [34]. Phosphorylation of AMPK attenuated hepatic steatosis and oxidative stress by increasing the phosphorylated expression level of ACC and down-regulating SREBP1 and SCD-1 protein expression. Importantly, 1-DNJ enhances energy metabolism and attenuates oxidative stress by activating the AMPK/SIRT1 pathway in db/db mice.

## 5 LIMITATIONS

In this study, it was found that 1-DNJ improved liver injury in db/db mice by regulating AMPK/SIRT1 pathway, but its specific regulatory mechanism still needs to be further clarified through overexpression and knockout of AMPK or SIRT1. 

## CONCLUSION

In conclusion, 1-DNJ attenuated liver lipid deposition and fibrosis of db/db mice by reduce TC, TG, LDL-C, AST, ALT, and TBiL, and its mechanism is related to attenuating the oxidative stress, reduce the protein expression of α-SMA, Collagen I, SREBP-1 and SCD-1, and upregulate the protein expression of SOD2, HO-1, NQO-1, p-AMPK/AMPK, p-ACC/ACC, and SIRT1 to enhancing the glucose-lipid metabolism.

## Data Availability

The data and supportive information are available within the article.

## LIST OF ABBREVIATIONS

SREBP1: Sterol Regulatory Element-Binding Protein 1
SCD-1: Stearoyl-CoA Desaturase 1
ACC: Acetyl-CoA Carboxylase
T2DM: Type 2 Diabetes Mellitus
NAFLD: Nonalcoholic Fatty Liver Disease
ALT: Alanine Aminotransferase
AST: Aspartate Aminotransferase
NASH: Nonalcoholic Steatohepatitis
