# Supplementary material for: 1-Deoxynojirimycin Ameliorates Diabetic Liver Injury by Regulating AMPK/SIRT1 and Oxidative Stress in db/db Mice
Source: Endocr Metab Immune Disord Drug Targets. 2025 Feb 19;22:E18715303327499. doi: 10.2174/0118715303327499250104221937 (PMC13555836; doi:10.2174/0118715303327499250104221937)
Supplement: Supplementary file 1 [file EMIDDT-26-E18715303327499_SD1.pdf]

Supplementary Material

1-Deoxynojirimycin Ameliorates Diabetic Liver Injury by Regulating AMPK/SIRT1 and Oxidative Stress in db/db Mice

Yao Li[1], Yu Cao[2], Yun-Yuan Tian[3], Wen-Wen Chen[2], Juan Wang[1], Meng-Meng Zhang[1], Si-WangWang[3] and Yan-Hua Xie[3,*]

[1]The College of Pharmacy, Shaanxi University of Chinese Medicine, Xianyang 712046, China; [2]Department of Chinese Materia Medica and Natural Medicines, Air Force Medical University, Xi'an 710032, China; [3]The College of Life Sciences, Northwest University, Xi'an 710069, China

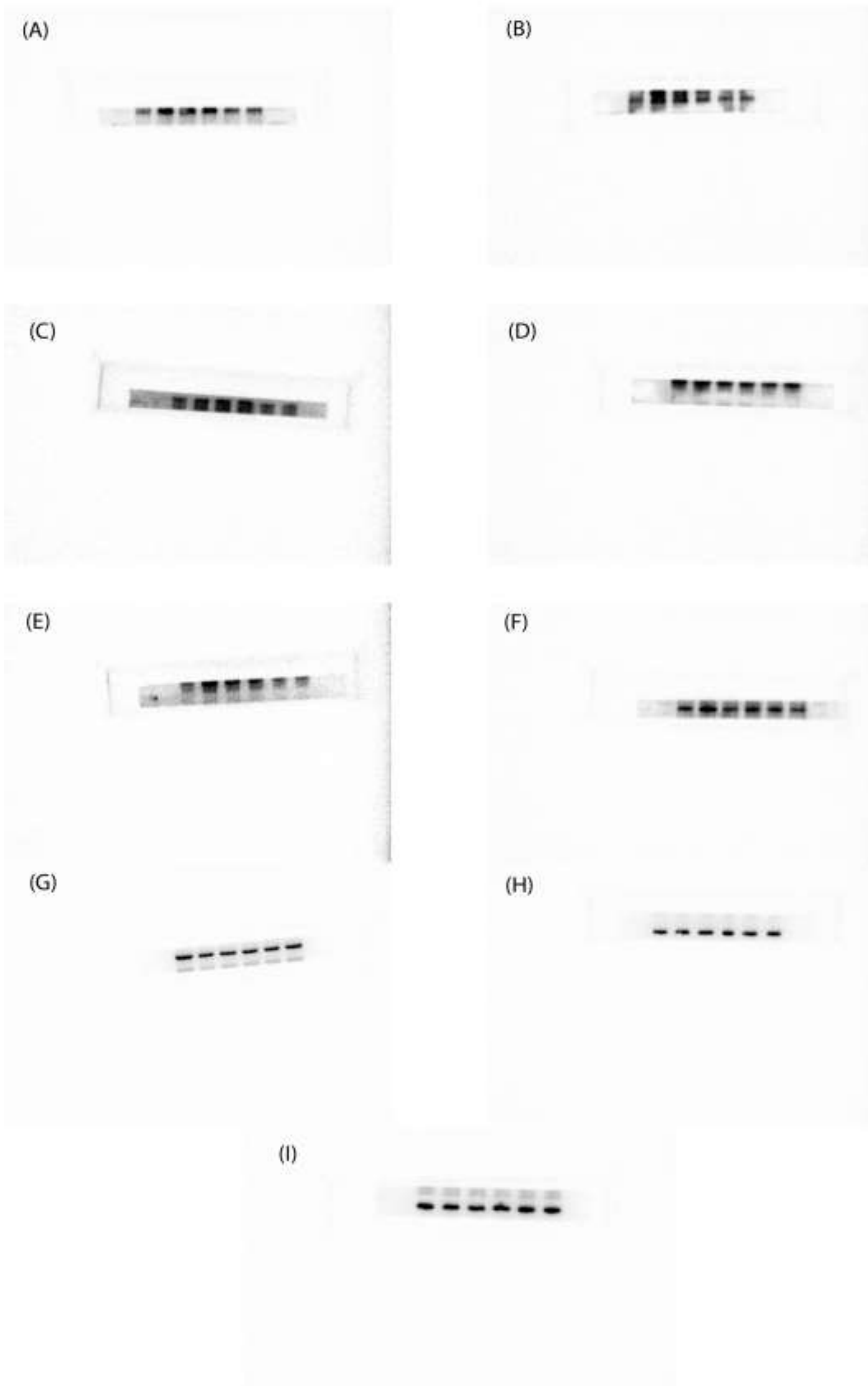

Fig. S1. The original data of Fig 3C. (A-C) Collagen I protein; (D-F) α-SMA protein; (G-I) β-actin protein.

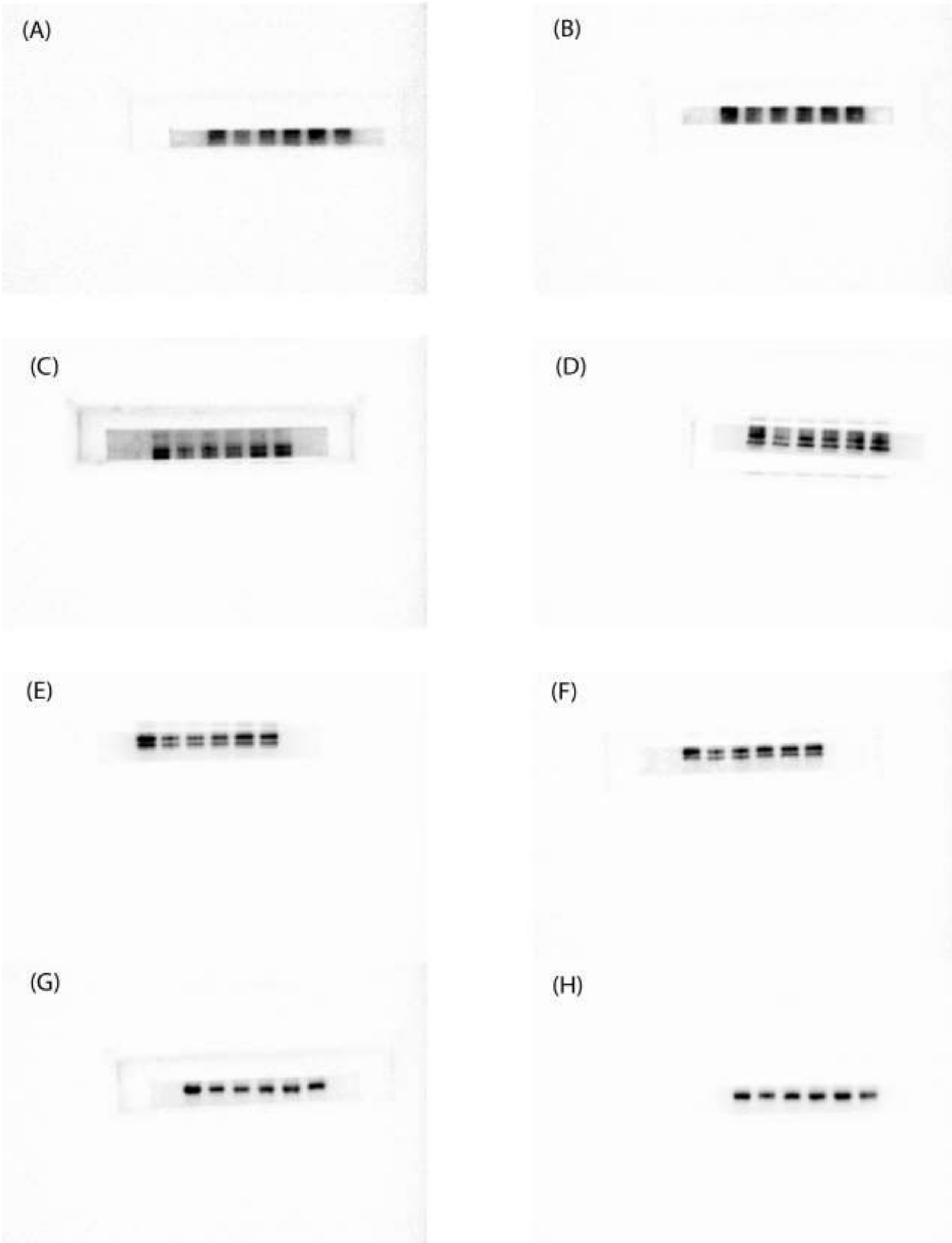
(A)
(B)
(C)
(D)
(E)
(F)
(G)
(H)

Cont….

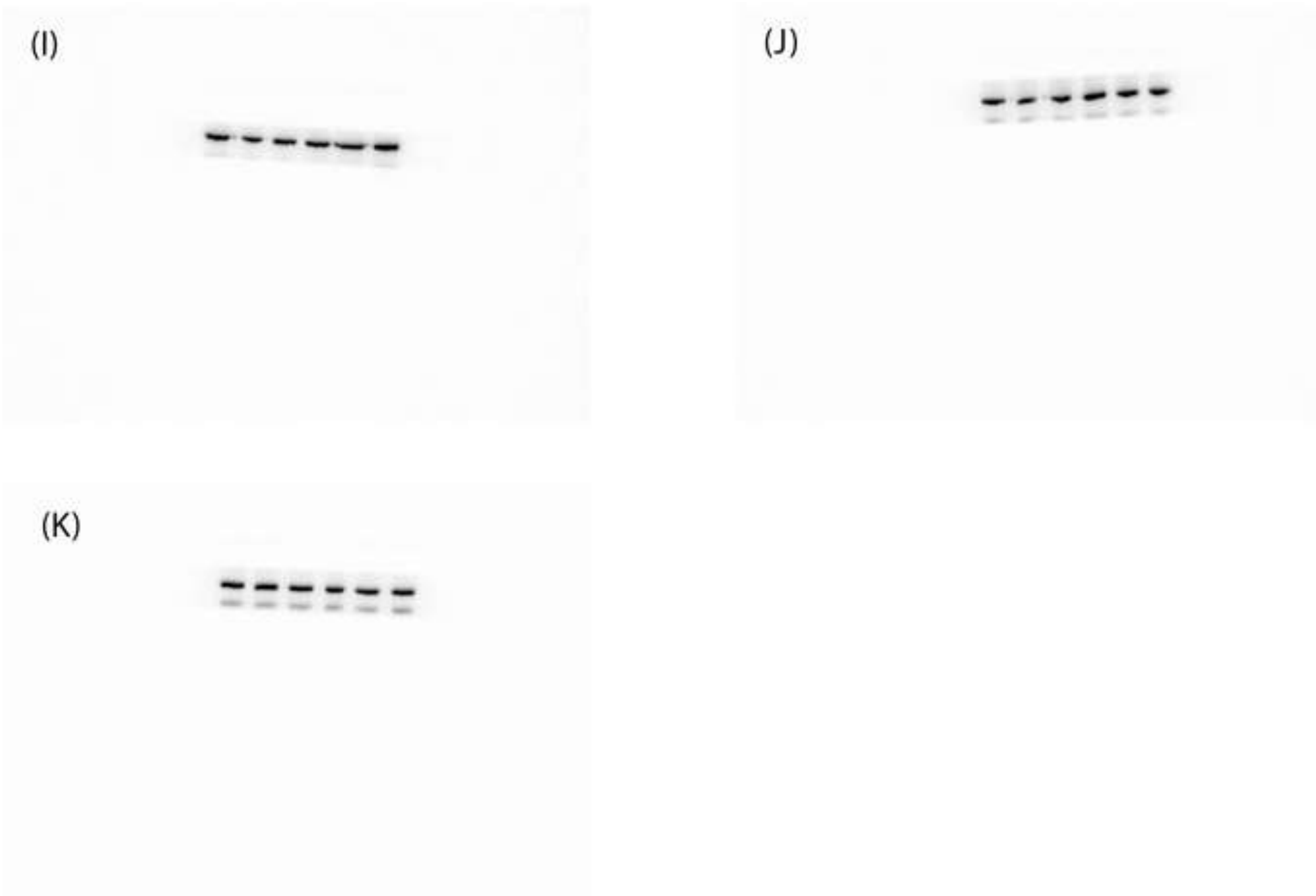

Fig. S2. The original data of Fig 4C. (A-C) HO-1 protein; (D-F) NQO-1 protein; (G-I) SOD protein; (J-K) β-actin protein.

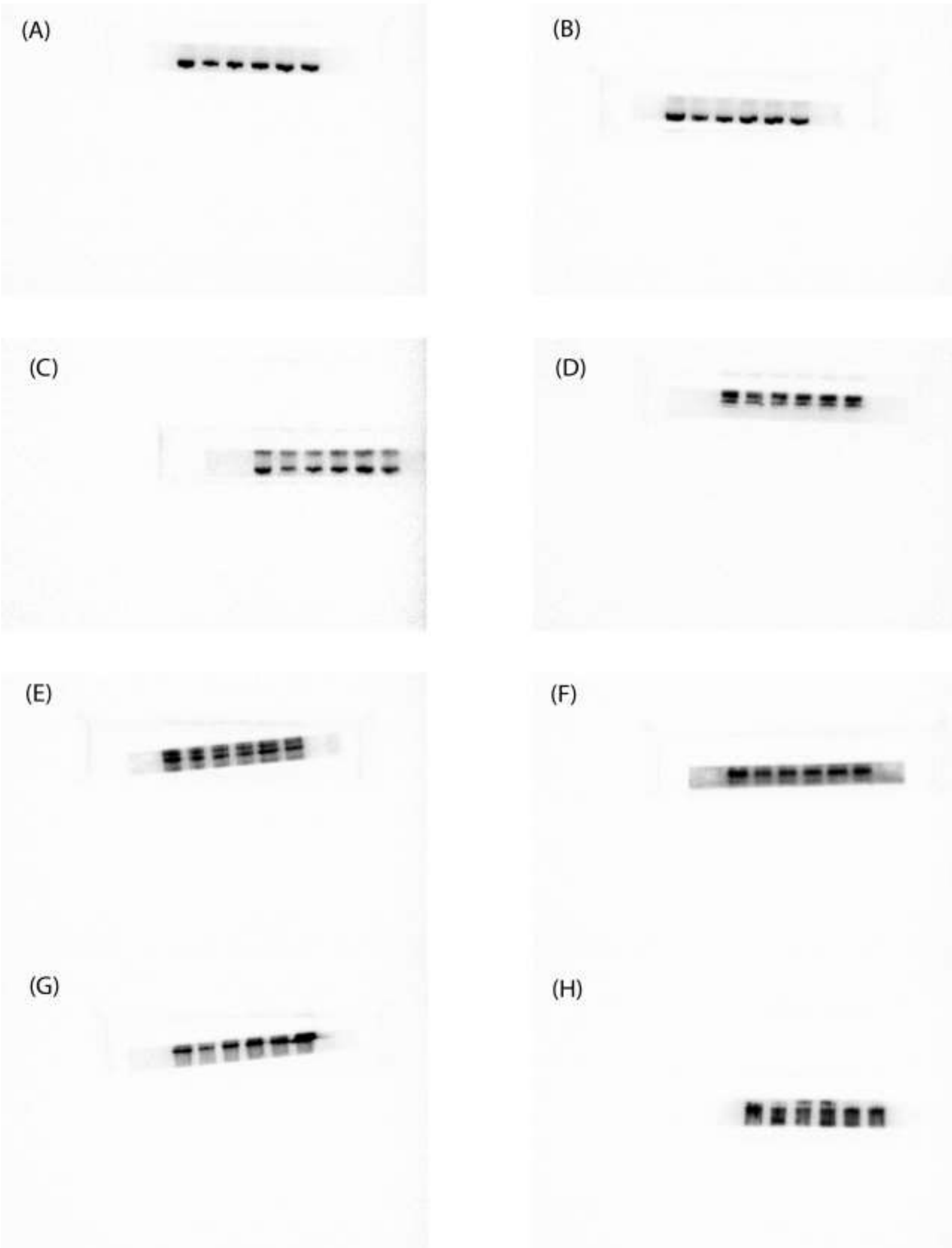

Cont...

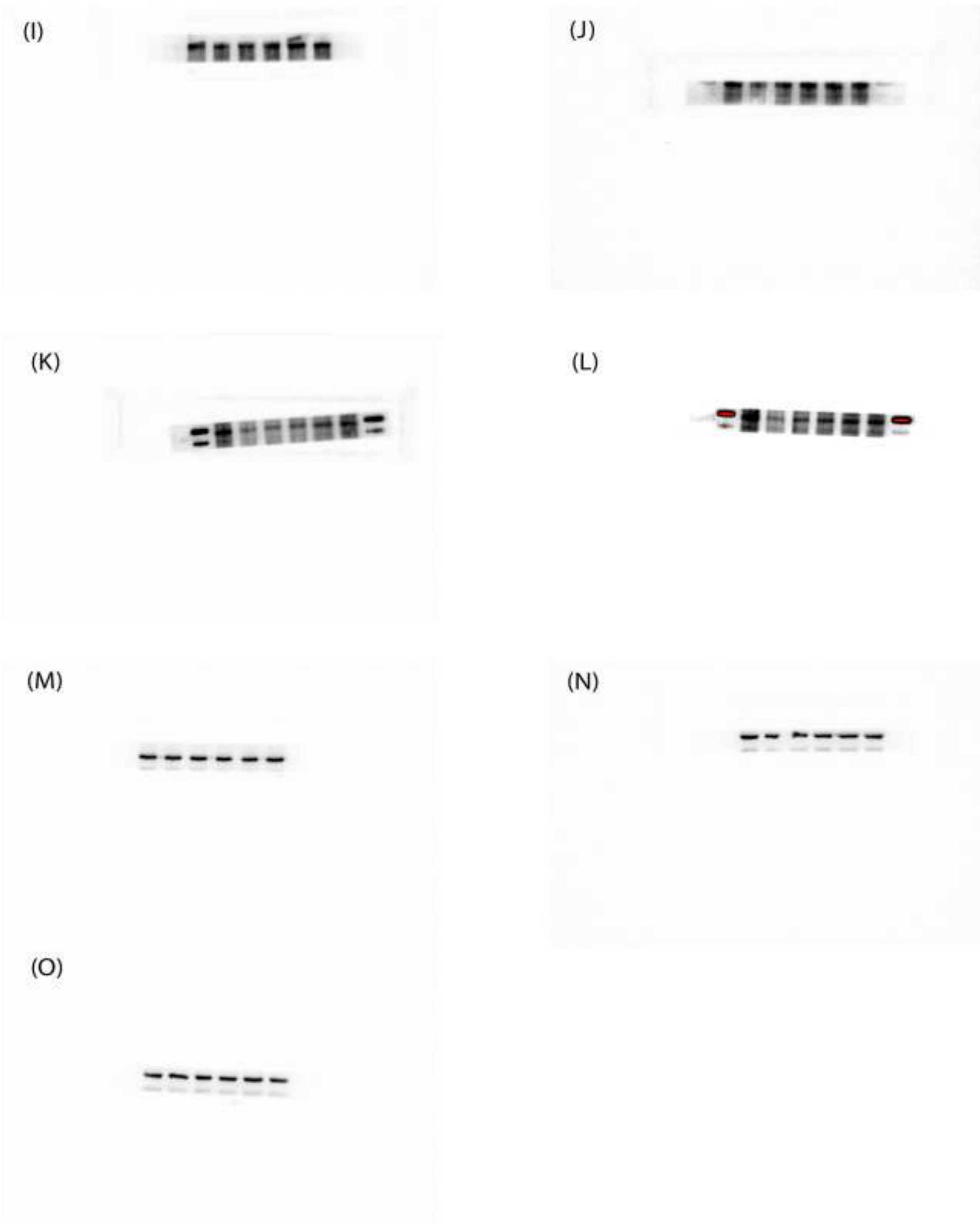

Fig. S3A. The original data of Fig 4A. (A-C) ACC protein; (D-F) AMPK protein; (G-I) p-ACC protein; (J-L) p-AMPK protein; (M-O) β-actin protein

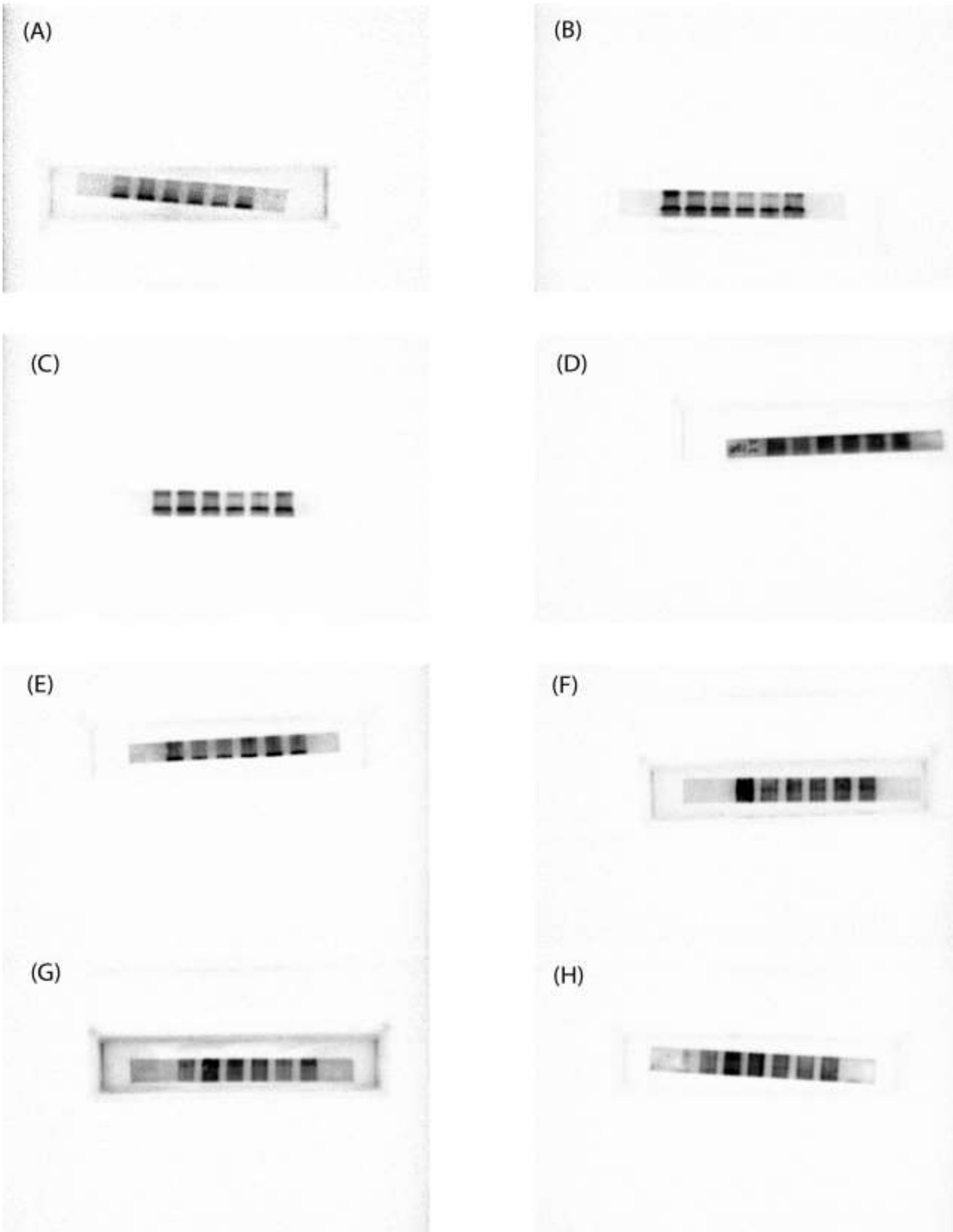

Cont…..

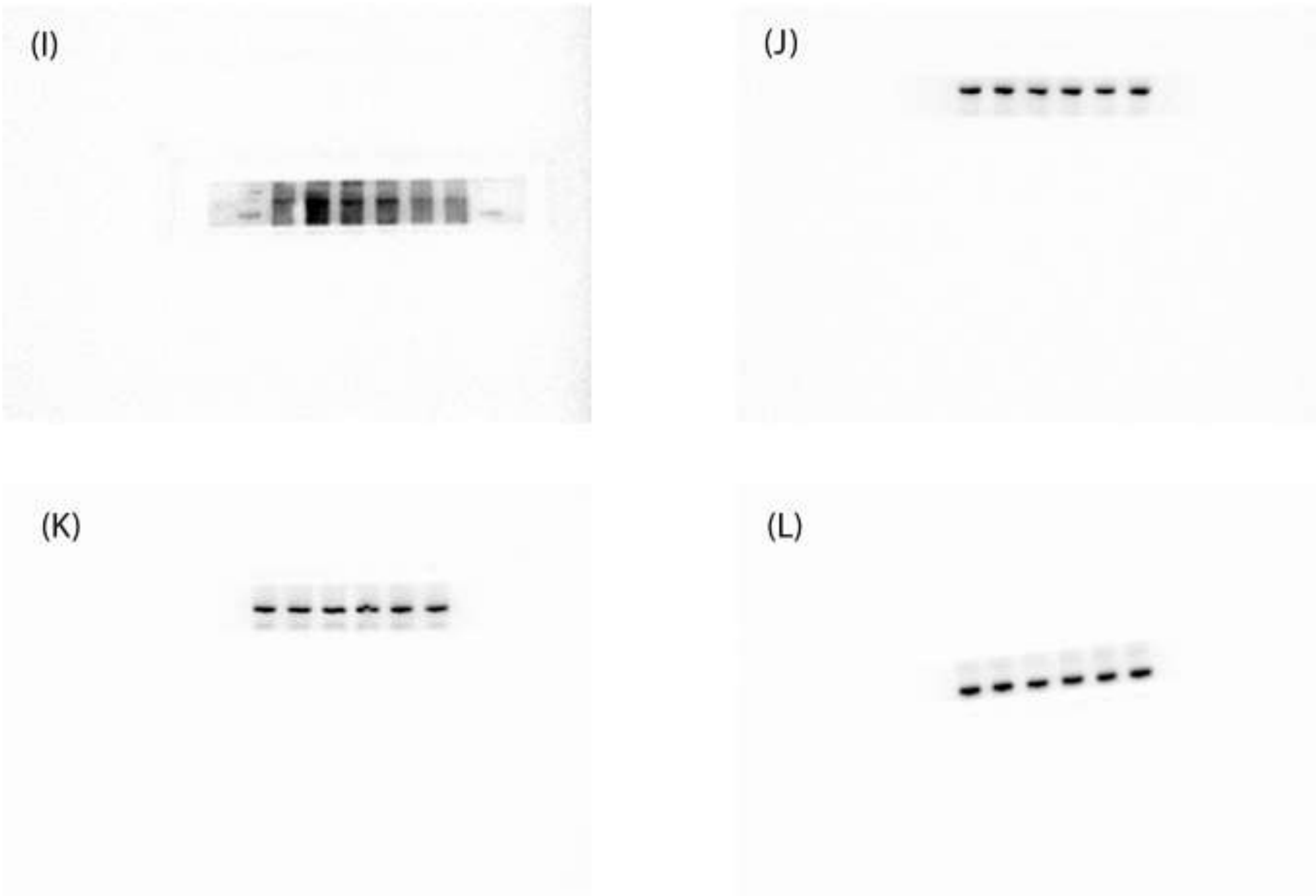

Fig. S3B. The original data of Fig 5B. (A-C) SCD-1 protein; (D-F) SIRT1 protein; (G-I) SREBP1 protein; (J-L) β-actin protein.
